# Versorgungswirksamkeit von Psychotherapie in Österreich

**DOI:** 10.1007/s00739-020-00686-w

**Published:** 2021-01-17

**Authors:** Henriette Löffler-Stastka, Markus Hochgerner

**Affiliations:** 1 Klinik für Psychoanalyse und Psychotherapie, Medizinische Universität Wien, Währinger Gürtel 18–20, 1090 Wien, Österreich; 2 Messerschmidtgasse 40/4, 1180 Wien, Österreich

**Keywords:** Vernetzung, Psychotherapeutische Versorgung, Angebots-Kette, Bedarf, Multiprofessionalität, Network, Psychotherapeutic care, Supply chain, Need, Multi-professionalism

## Abstract

Die aktuelle Versorgungssituation psychisch Kranker in Österreich wird zur Diskussion gestellt. Dabei steht eine Analyse der Versorgungswirksamkeit von Psychotherapeutinnen und Psychotherapeuten in freier Praxis im Vordergrund. Anhand eines Fallbeispiels wird die Wichtigkeit der interdisziplinären Zusammenarbeit erläutert.

## Abstract (en)

The current health care situation of people with mental health problems is discussed. The focus of the discussion is an analysis of the effectiveness of care provided by psychotherapists in private practice. The importance of interdisciplinary cooperation is explained using a case study.

## Versorgungsnotwendigkeit und Versorgungsvolumen

Psychische Erkrankungen wie Angst, Angst und Depression gemischt sowie somatoforme Störungen haben somatische Erkrankungen im Ranking der häufigsten Krankheitsbilder der Österreicher 2019 abgelöst. Laut OECD und europäischer Kommission 2018 („Gesundheit auf einen Blick“) wurde bereits 2014 festgehalten, dass psychische Erkrankungen rund 3,5 % des österreichischen Bruttoinlandsproduktes (BIP), somit 11 Mrd. Euro Kosten und größtes individuelles und familiäres Leid erzeugen [3]. Dieser Betrag hat sich bis 2019 auf 4,3 % des BIP, somit 13,9 Mrd. Euro gesteigert. Im Rahmen der COVID-19-Folgeerkrankungen wird eine aktuelle weitere Steigerung um 20 % der genannten Krankheitsbilder in ersten Erhebungen sichtbar.

Langzeitstudien ergeben eine Prävalenz von 25–30 % psychischer Symptome und Erkrankungen im Lebenslauf [7], jedoch nur 30 % der Betroffenen werden im kassenfinanzierten Hilfssystem vorstellig.

Die Bereitschaft, psychotherapeutische Hilfe in Anspruch zu nehmen, wäre mit guter Versorgungsstruktur bei der Hälfte der Erkrankten erreichbar. Gehen wir von einer jährlichen Bedarfszahl von (je nach Untersuchung) 3–7 % der Gesamtbevölkerung aus, entstünde ein Bedarf bei 1,5–3 % der Bevölkerung, somit ca. 125.000–250.000 Patienten jährlich bei einer durchschnittlich erwartbaren Behandlungsdauer von 25–40 Einheiten. In Summe werden derzeit unter Einbezug aller psychotherapeutischen und psychotherapienahen Versorgungsformen ca. 100 Mio. Euro für Psychotherapie jährlich ausgeschüttet. Die Inanspruchnahme von Psychotherapie bei erfassten 1,5 % der Bevölkerung ergibt jedoch ein Finanzierungsvolumen von jährlich mindestens 260 Mio. Euro bei sehr nieder eingeschätztem Honorar von 77.- Euro je Stunde Psychotherapie [5].

## Gesetzliche Grundlage für psychotherapeutische Versorgung

Psychotherapie wurde mit dem Bundesgesetz 361/1990 als Berufsbild mit der Qualifikation zur eigenständigen Behandlung psychischer, psychosozial und psychosomatisch bedingter Leidenszustände anerkannt. Wurden zum damaligen Zeitpunkt 1650 Psychotherapeutinnen und Psychotherapeuten als berufsberechtigt erfasst, sind aktuell mit 01.06.2020 bereits 10.415 Personen in die Berufsliste des für Gesundheit zuständigen Ministeriums eingetragen [4] und stellen einen wesentlichen Beitrag zur Versorgung psychisch Leidender in Österreich dar.

## Behandlungsangebot und finanzieller Rahmen

Durchschnittlich werden pro Therapeut 12 Wochenstunden Psychotherapie angeboten – davon 46 % in freier Praxis. Zur Arbeit in Institutionen fallen die regional besonders unterschiedlichen Zahlen auf: Während in Wien 19,3 % der Therapeuten auch in Institutionen arbeiten, sind in den Bundesländern NÖ, OÖ und ST nur noch 9,6 bis 6,2 % in Einrichtungen des Gesundheitswesens tätig. Es folgen ST, TI mit 6,2 bis 5,0 %, Schlusslichter sind VB, KÄ, BU mit 2,4–1,4 %. Etwa ein Drittel der Personen je Bundesland sind sowohl institutionell als auch freiberuflich tätig [6].

Stundensätze oft ohne ausreichende Rückerstattungsmöglichkeit für Patienten

Im Jahr 2019 wurden seitens der Sozialversicherung für Psychotherapie im engeren Sinne 76,4 Mio. Euro ausgegeben. Davon entfielen 60 % auf durch Versorgungsvereine und Institutionen angebotene Psychotherapie, 20 % auf Vertrags- und Wahlärzte, 19 % auf niedergelassene Therapeuten und 1 % auf Leistungen kasseneigener Einrichtungen [2].

In Summe wurden dabei durch Psychotherapeuten 116.000 Personen erreicht, die ärztlichen Leistungen psychotherapeutischer Medizin versorgten 117.000 Personen (grober Richtwert durch Fehlen weiteren Datenmaterials). Durchschnittlich wurden 20 Therapiestunden in Anspruch genommen. „Das vorhandene Angebot stellt eine Basisversorgung sicher, dennoch scheint in allen Bundesländern weiterer Versorgungsbedarf gegeben“ und es „kann davon ausgegangen werden, dass das vorhandene Angebot den Bedarf nicht deckt“ (ebendort, S. IV) [2].

## Bezahlung und Kostenzuschüsse

Im Jahr 1992 wurde in der 50. ASVG-Novelle (BGBL 676/1991) Psychotherapie der ärztlichen Tätigkeit gleichgestellt und in den Pflichtleistungskatalog der sozialen Krankenversicherung aufgenommen. Es fehlt jedoch bislang ein Gesamtvertrag. Damit werden die heterogene und je Bundesland unterschiedliche Form und der Umfang der Bezahlung ein wesentliches Moment der Versorgungswirksamkeit: Laut Statistik der Gesundheit Österreich GmbH (GÖG) [6] wird überwiegend Kostenzuschuss gewährt (52,2 % Rückerstattung von Teilkosten mit 28.- bis 50.- Euro pro Stunde Sachleistung) oder eine Vollrefundierung durch die Kassen (26,8 % der Patienten) getragen. Psychotherapie über Selbstzahlung (21 %) erfolgt bei durchschnittlichen Stundensätzen von 80.- bis 120.- Euro. Hinzu kommen ca. 4500 Psychotherapeuten, die im Ausbildungskontext unter Supervision eine ähnlich hohe wöchentliche Versorgungsfrequenz wie berufsberechtigte Therapeuten aufweisen, jedoch noch ohne Möglichkeit zur Abrechnung mit den Kassen und meist mit einem fragwürdigen Stundensatz von 10.- bis 40.- Euro ohne Rückerstattungsmöglichkeit für Patienten.

## Versorgungswirksamkeit und Hindernisse

Als „versorgungswirksam“ werden lt. GÖG alle Therapeuten eingestuft, die Patienten mit krankheitswertiger Störung versorgen [6]. Dabei wird deutlich, dass zu 26,1 % schwere und zu 43,8 % mittelgradige Störungen erfasst werden. Die Versorgung der unterschiedlichen Altersgruppen zeigt Spezialisierungen mit einem eigenständigen Weiterbildungsausweis am zuständigen Bundesministerium für Gesundheit in den Bereichen Säuglinge/Kinder (18,1 %) sowie Jugendliche (34,8 %) und ältere/alte Menschen (34,7 %).

Patientenstatement aus der Inanspruchnahme-Erhebung: „Am hilfreichsten war die Kontinuität“

Als besonderes Manko in der Versorgungswirksamkeit zeigen sich die weiter bestehende Stigmatisierung psychischer Fragestellungen, die schwierige und bürokratische Antragstellung mit Wartezeiten bis zu mehreren Monaten, Informations- und Kommunikationsdefizite, die mangelnde Möglichkeiten zur psychosozial vernetzten Betreuung in einer geschlossenen Versorgungskette mit psychiatrischen und psychosomatischen Angeboten und die ethisch fragwürdige jährliche Kontingentierung der gewährten Stundenzahl je Bundesland [5].

## Hilfe durch geschlossene Versorgungsketten

Diese aufschlussreiche Studie [5] mit quantitativen und qualitativen Elementen zeigt die Bedeutung der Motivation durch die soziale Umgebung und die besondere Rolle der Hausärzte in der Zuweisung zur Psychotherapie und zu den medizinischen Fachdiensten, die in gelungener Abfolge eine hilfreiche und zeitgerechte Zuweisung zu stationären Angeboten mit psychotherapeutisch weiterführender Therapie auch eine wesentliche Maßnahme der Tertiärprävention zur Vermeidung weiterer Chronifizierung, Krisenhaftigkeit und akutstationärer Aufenthalte darstellt.

**Figure 1:**
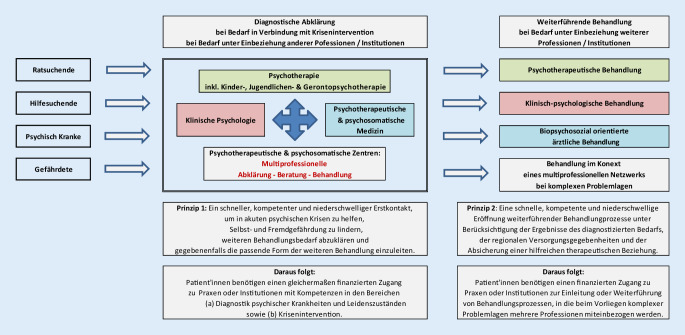

Zusätzlich ist eine Vernetzung im multiprofessionellen Team von wesentlicher Bedeutung. Wie ein solches Versorgungsnetz gestaltet sein kann und welche handelnden Berufsgruppen hier effizient zusammenarbeiten müssen, ist in Abb. 1 dargestellt (siehe auch [1]).

Der Aufbau einer multiprofessionellen Versorgungsstruktur in Österreich zur diagnostischen Abklärung und Behandlung von psychischen und psychosomatischen Krisen und Erkrankungen umfasst die in Abb. 1 dargestellten Berufsgruppen. Bei Bedarf kann die Versorgung unter Einbeziehung weiterer Professionen und Institutionen aus Bereichen wie Sozialarbeit, Schuldnerberatung, Erziehungsberatung, Physiotherapie, Ergotherapie, Musiktherapie, Frühe Hilfen, Frühförderung, Psychagogik im Kontext von Schule etc. auf der Basis öffentlicher Finanzierung erfolgen (vgl. [1]).

## Biopsychosoziale Versorgung – konsequente Vernetzungsarbeit

Anhand des folgenden Fallbeispiels zur Diagnostik einer Persönlichkeitsstörung und Suchterkrankung wird die Wichtigkeit eines psychosozialen Netzwerkes deutlich.

Vernetzungsarbeit erfordert spezifische Kompetenzen

Diese Vernetzungsarbeit erfordert spezifische Kompetenzen: die Fertigkeit, eine psychiatrische Diagnostik und Differentialdiagnostik nach ICD-10/11 durchzuführen, einen Überblick über Klassifikationsschemata zu haben, um die Persönlichkeit der Patientin einzuschätzen, damit individuell abgestimmt zu intervenieren und auch prognostisch wesentliche Informationen ableiten zu können. Kenntnisse der operationalisierten psychodynamischen Diagnostik (OPD) und weitere Formen psychotherapeutischer Diagnostik können von Vorteil sein. Innerhalb der psychotherapeutischen Diagnostik ist für diesen Fall das Erstellen einer Strukturdiagnose (anhand der Beurteilung von Realitätsprüfung, Identität, Abwehrmechanismen) wesentlich. Allgemein zählen die Abklärung der Therapiemotivation, der Behandlungsvoraussetzungen und das Erheben der subjektiven Krankheitstheorie zu den Kernkompetenzen der psychotherapeutisch Ausgebildeten. Kenntnisse der Persönlichkeitsstörung, Unterschiede zwischen psychiatrischer Diagnostik und psychotherapeutischer Diagnostik sowie Therapieplanung und Abfassen eines Patientenbriefes sind hier wesentlich, um adäquat versorgen zu können.

Zugewiesen von einem Allgemeinmediziner oder Psychotherapeut könnte nach erfolgter psychiatrisch/psychotherapeutischer Diagnostik ein Patientenbrief wie folgt aussehen.

## Patientenbrief

(Von einem psychiatrisch/psychotherapeutischen Fachexperten an den Allgemeinmediziner).

Sehr geehrte Frau Kollegin! Sehr geehrter Herr Kollege!

Wir erlauben uns – mit Einverständnis und Wissen der Patientin – über Patientin A. zu berichten, die von Ihnen an unsere Klinik zur Psychotherapieplanung zugewiesen wurde. Die Patientin wird von Ihnen im Rahmen des Drogen-Substitutionsprogrammes behandelt. Es wurden ein psychotherapeutisches Erstgespräch und ein strukturelles Interview durchgeführt.

### Zuweisungsmodus:

Die Patientin betont einerseits, aus Eigeninitiative an die Klinik zu kommen, andererseits berichtet sie in ambitendenter Art und Weise, dass sie die Möglichkeit zur diagnostischen Abklärung und Psychotherapieplanung von ihrem praktischen Arzt erfuhr und von diesem auch „hierher geschickt“ wurde.

### Beschreibung der Patientin:

Die Patientin, 28 Jahre alt, untergewichtig, unsicher im Auftreten, wirkt vordergründig kooperativ, unterwürfig, leicht irritierbar, während des Gespräches droht die Patientin ständig einen Wutausbruch zu entwickeln, bricht beispielsweise in Tränen aus, versucht Mitleid zu erwecken. Dies alles wirkt jedoch künstlich, gekünstelt und unecht.

### Lebensgeschichte:

Die Patientin sei etwa vier Wochen zu früh auf die Welt gekommen, habe die erste Zeit von der Mutter getrennt im Inkubator verbracht, sei nicht gestillt worden.

Der Vater sei wenig anwesend gewesen, die Mutter sei immer Hausfrau gewesen, habe nach der Pflichtschule keinerlei weitere Ausbildungen absolviert. Zur Hochzeit der Eltern sei es nur wegen des unerwarteten Kindes und auf Druck der jeweiligen Eltern gekommen.

Die Patientin beschreibt eine konfliktreiche Ehe ihrer Eltern: Die Mutter sei alkoholabhängig gewesen, Mutter und Vater hätten zahlreiche außereheliche Beziehungen gehabt. Sie habe nächtelang den Streitereien der Eltern zuhören müssen, dabei sei es auch regelmäßig zu Handgreiflichkeiten gekommen.

Mit 15 Jahren erster Kontakt mit Drogen, über mehrere Jahre auch regelmäßiger intravenöser Konsum.

Nach Abbruch des Gymnasiums habe sie mit Gelegenheitsarbeiten begonnen, dabei häufige Wechsel der Arbeitgeber.

Die Patientin hat drei Kinder aus jeweils drei verschiedenen Partnerschaften. Von allen Männern habe sie sich nach einigen Monaten getrennt. Sie lebe derzeit in einer konflikthaften Beziehung zu einem gehbehinderten Mann, der eine Erwerbsunfähigkeitspension beziehe.

### Beschwerden:

Die Patientin beschreibt, dass sie schon als Kind ihre Stimmung und Befindlichkeit „wie hinter einem grauen Schleier verhüllt“ erlebt habe. Sie habe jahrelang Drogen konsumiert (auch während der Schwangerschaft; derzeit konsumiere sie in der Wohnung Cannabis) und mache sich Vorwürfe, ihren Kindern keine gute Mutter sein zu können. Ihr Ziel sei es, von dem gegenwärtig verordneten Substitutionsmittel wegzukommen und völlig abstinent zu leben.

### Einstellung der Patientin zu ihren Beschwerden:

Die Patientin macht die Mutter für alle ihre Probleme verantwortlich. In der Auseinandersetzung mit der Mutter habe die Patientin beispielsweise gelernt, Drogen zur Stimmungs- und Konfliktregulation einzusetzen. Sie macht die konfliktbeladene Atmosphäre in ihrer Kindheit für ihre Probleme verantwortlich.

### Selbstrepräsentanzen:

Sie sei eigentlich ein positiver, sehr interessierter Mensch (Sprachen, Gitarre spielen, mit den Kindern spielen), sie werde in der Arbeit geschätzt und gemocht, könne aber von der Vergangenheit nicht loslassen, achte sehr darauf, wie sie auf andere wirke und vergesse dabei, den anderen überhaupt wahrzunehmen. Sie habe eine dauernde innere Anspannung.

### Vorstellung von wichtigen Beziehungspersonen (Objektrepräsentanzen):

Zu ihren Eltern habe sie keine vertrauensvolle Beziehung gehabt, vor allem der Kontakt zur Mutter sei von Geburt an gestört gewesen, auch die Mutter habe Schwierigkeiten mit der Tochter gehabt. Vor drei Jahren seien beide Eltern (beide an Karzinomen) und die Großmutter (an Altersschwäche) verstorben.

Ihre jüngste Tochter koche gerne, möchte später ein Gasthaus eröffnen, sei harmoniebedürftig und möchte immer alle einbinden. Sie sei sehr fürsorglich und trage eine innere Traurigkeit in sich.

### Nosologische und psychodynamische Krankheitshypothese:

Identitätsdiffusion, Abwehr: Spaltung, Projektion, projektive Identifizierung, Idealisierung und Entwertung; Realitätsprüfung erhalten, paranoider Verarbeitungsmodus, emotionale Instabilität; die Patientin tendiert dazu, anfangs jegliche Kooperation zu bejahen (Idealisierung), um zu einem späteren Zeitpunkt das jeweilige Vorhaben wieder infrage zu stellen (Entwertung).

### Diagnose:

ICD-10: F12.2, F60.31.

Strukturelle Diagnose: Borderline-Persönlichkeitsorganisation.

### Procedere:

Wir empfehlen nach testmäßiger Erfassung des kognitiven Leistungsniveaus, Ausschluss einer Alkoholembryopathie und Spezifizierung des Motivationsstadiums der Patientin eine psychoanalytisch orientierte Psychotherapie, da die Patientin in gravierenden Bereichen ihrer Charakterstruktur so beeinträchtigt ist, dass eine rein symptomorientierte Behandlung nicht zielführend erscheint. Die mehreren Psychotherapieversuche, die bereits gemacht wurden und misslungen sind, erfordern eine enge Kooperation mit Ihnen als Allgemeinmediziner sowie Ihre Unterstützung der Behandlung. Eine grundlegende Arbeit an der Strukturierung der Persönlichkeit, insbesondere angesichts der Jugend der Patientin, erscheint sinnvoll. Wir ersuchen Sie, weiterhin längerfristig kooperativ mit uns (und dem behandelnden Psychotherapeuten) weiterzuarbeiten, da die der Erkrankung inhärenten Spaltungsprozesse möglicherweise immer wieder die Behandlung durch einen Abbruch bedrohen können.

## Fazit für die Praxis

- Psychotherapeutische Hilfe wird von 1,5–3 % der Bevölkerung in Anspruch genommen.
- Je zur Hälfte werden die Betroffenen durch Psychotherapeutinnen und Psychotherapeuten sowie durch die ärztlichen Leistungen psychotherapeutischer Medizin erreicht.
- Psychotherapeutinnen und Psychotherapeuten in freier Praxis versorgen zu 26,1 % schwere und zu 43,8 % mittelgradige Störungen.
- Durchschnittlich werden pro Therapeut 12 Wochenstunden Psychotherapie angeboten – davon 46 % in freier Praxis.
- Es besteht ein deutlicher Mangel an Möglichkeiten zur biopsychosozial vernetzten Betreuung in einer geschlossenen Versorgungskette mit individuell abgestimmten Therapien in Dosis und Zeit.
- Eine gut abgestimmte multiprofessionelle Zusammenarbeit unter Beachtung und Kenntnis der unterschiedlichen Kompetenzen ist erforderlich.
